# Perceptions on the risk communication strategy during the 2013 avian influenza A/H7N9 outbreak in humans in China: a focus group study

**DOI:** 10.5365/WPSAR.2016.7.1.005

**Published:** 2016-07-11

**Authors:** Richun Li, Ruiqian Xie, Chong Yang, Melinda Frost

**Affiliations:** a United States Centers for Disease Control and Prevention, Beijing, People’s Republic of China.; b Chinese Center for Health Education, Beijing, People’s Republic of China.; c United States Centers for Disease Control and Prevention, Beijing, People’s Republic of China.

## Abstract

**Objective:**

To identify the general public’s perceptions of the overall risk communication strategy carried out by Chinese public health agencies during the first wave of avian influenza A(H7N9) outbreak in humans in 2013.

**Methods:**

Participants were recruited from communities in Beijing, Lanzhou and Hangzhou, China in May and June 2013 by convenience sampling. Demographics and other relevant information were collected using a self-administered questionnaire. Focus group interviews were conducted using a set of nine pre-developed questions and a tested moderator guide. The interviews were audio recorded and were transcribed verbatim. The constant comparative method was used to identify trends and themes.

**Results:**

A total of nine focus group interviews, with 94 participants recruited from nine communities, were conducted. Most participants received H7N9 information via television and the Internet. Most the participants appreciated the transparency and timeliness of the information released by the government. They expressed a sense of trust in the recommended public health advice and followed most of them. The participants suggested that the government release more information about clinical treatment outcomes, have more specific health recommendations that are practical to their settings and expand the use of new media channels for risk communication.

**Conclusion:**

The public perceived the overall risk communication strategy by the Chinese public health agencies as effective, though the moderator had a governmental agency title that might have biased the results. There is a need to expand the use of social media for risk communication in the future.

## Introduction

Effective risk communication is an essential element for outbreak management and health emergency response for pandemics. (*1*, *2*) Successful risk communication should (1) instruct, inform and motivate self-protective behaviour; (2) update risk information; (3) and build trust. (*3*) Based on previous experience in handling disease outbreaks with pandemic potential, risk communication strategies in China have evolved in the last decade. After the 2003 severe acute respiratory syndrome (SARS) outbreak, the Chinese government’s awareness of and capacity to respond to health emergencies substantially improved. (*4*) China established a new mechanism for emerging infectious disease response with improvements in command and decision-making, organization and collaboration, monitoring and early warning and protection and communication. This new mechanism allowed China to successfully manage the avian influenza H5N1 and the pandemic influenza A/H1N1 outbreaks in 2005 and 2009, respectively. (*5*)

Human infection with the avian influenza A(H7N9) virus were first identified in China in March 2013. (*6*) H7N9 is a strain of influenza that causes mild disease in poultry but can be severe in humans. The World Health Organization reported 133 cases in the first wave of the H7N9 outbreak in China from February to May 2013; however, the number of cases decreased in the following summer. (*7*) There is no vaccine to prevent human infection with H7N9 virus, and population immunity was low for this novel virus. Human-to-human transmission of H7N9 was uncertain at the early stage of the outbreak, and there was much concern that human infection with H7N9 virus could rapidly spread the disease, resulting in a pandemic threat. (*8*, *9*) Given that concern, in this study we conducted focus group interviews in three cities in China to assess China’s risk communication responses to the 2013 H7N9 outbreak in humans from the general public’s perspective.

## Methods

### Study design

Focus group analysis was used to gain qualitative data on audience perceptions, feelings and opinions about health information provided during the outbreak. (*10*, *11*) Prior to the interviews, participants were also requested to complete a short self-administered questionnaire which collected demographic information, awareness of H7N9 and major channels through which the participants received or sought H7N9 information.

### Study sites

To achieve a reasonable representation of humans infected with H7N9 virus in China, we selected Beijing, Hangzhou (capital city of Zhejiang Province) and Lanzhou (capital city of Gansu Province) for the study. These cities represent areas with low human H7N9 case numbers, high case numbers and no identified cases, respectively, as well as different geographic locations in China. As of 31 May 2013, the number of human H7N9 cases in Shanghai city, Zhejiang Province and Jiangsu Province in east China accounted for 81.5% of the total number of cases. (*12*) Hangzhou reported 30 confirmed cases; (*13*) two cases were identified in Beijing (*14*) but no cases were reported from Gansu Province.

### Study participants

According to the general focus group planning strategies,10 we decided the size of the focus group to be

8–13 individuals for ample discussion. We conducted three focus group interviews in each city to reach information saturation. Subjects were recruited by convenience sampling. Inclusion criteria were people who were aged 16 years or above, resided in the community and had normal oral conversation ability. Eligible individuals were invited by community committee workers through telephone or face-to-face communications to participate in the study. Subjects were then randomized into different focus groups according to the order of recruitment. Specific occupational groups such as health-care workers or poultry workers did not have higher priority in the recruitment process.

### Focus group interviews

The interviews were conducted in May and June 2013 at local community facilities (e.g. community residents’ activity centres and community health centres) easily accessed by the participants. Each participant received information about the objectives and procedures of the study and signed a consent form before participating. All interviews were run by one experienced moderator following a tested moderator guide with nine major questions (Table 1). The moderator guided the discussion by asking pre-developed, open-ended questions and encouraged all the participants to contribute opinions by using probes. The questions were arranged in the order of introductory question (question 1), which normally is the easiest question for everyone to answer, transition question (question 2), key questions (questions 3–8) and ending question (question 9). (*15*) The interviews lasted from 60 to 90 minutes and were audiotaped with the consent of all the participants. While the interviews were being audio recorded, the interviewees’ identities were ensured to be anonymous. The moderator had experience conducting many focus group interviews with the Chinese public on various public health issues as well as having expert knowledge in risk communication. Participants were allowed to quit the study at any time without giving any reasons. Each participant received an incentive of 50 yuan (equivalent to approximately US$ 8) after the interview.

**Table 1:** Open-ended focus group interview questions, influenza A/H7N9 perception study, China, 2013

Questions
**1. Have you previously heard of H7N9 avian influenza?**
**2. Do you worry about H7N9 avian influenza? Why?**
**3. What information do you feel you need the most about H7N9 avian influenza?**
**4. What information sources do you consider to be credible?**
**5. What H7N9 related information released by government is most helpful for you?**
**6. What public health recommendations have you adopted? Why?**
**7. Are you satisfied with the government’s information release and communication practice?**
**8. In terms of the government’s information release and health education relevant to H7N9, what aspects do you consider to be good practice? Why?**
**9. What recommendations do you have to improve the government’s information release and communication practice about H7N9?**

### Data analysis

The data from the self-administered questionnaire were analysed using SAS (SAS 9.3, Cary, NC). Use of social media/Internet was defined as use of short message service (SMS), web portals (e.g. Baidu), microblogging (e.g. Sina Weibo) and WeChat (a mobile instant text messaging communication application) for information on H7N9. The audio files were transcribed verbatim from the focus group interviews. The transcripts were reviewed and coded by the first author (who was also the assistant moderator and field note taker). The constant comparative method (*10*) was used to identify trends and themes. The team had summary discussion after each interview to reach preliminary consensus of key findings.

### Ethics

The proposal of this study was submitted to the Center for Global Health (CGH) of Centers for Disease Control and Prevention for project determination and approval. It was deemed as “not human subjects research” and “does not require human subject research review beyond CGH.”

## Results

In total, 145 eligible individuals were approached and 94 participants were recruited to this study.

The response rate was about 65%. The majority were female (76.6%), aged 30 years or above (71.3%), and more than half of them (56.4%) had a high school education level or below. Participants were evenly distributed across the three cities (Table 2). Awareness of the H7N9 outbreak was very high due to broad media coverage, and most participants reported that they received their first information about the H7N9 outbreak during late March to early April 2013 via TV (67.0%), Internet/social media (48.8%) or newspaper (37.2%) (Table 3).

**Table2:** Demographics of the study participants, influenza A/H7N9 perception study, China, 2013 (*n* = 94)

Characteristics	N	%
**Gender**		
Male	22	23.4
Female	72	76.6
**Age (years)**		
16–30	27	28.7
31–50	34	36.2
51–73	33	35.1
**Education**		
Middle school and below	26	27.7
High school	27	28.7
College and above	41	43.6
**City**		
Beijing	33	35.1
Hangzhou	34	36.2
Lanzhou	27	28.7

**Table 3:** How participants first heard of influenza A/H7N9 and where they searched for more information, influenza A/H7N9 perception study, China, 2013*

Information source	First heard of influenza A/H7N9 *n*(%)	Searched for more information *n*(%)
**TV**	63(67.0)	49(62.8)
**Radio**	9(9.6)	5(6.4)
**Newspaper**	35(37.2)	27(34.6)
**Web portal**	18(19.1)	44(56.4)
**Weibo**	13(13.8)	0
**Search engine**	3(3.2)	0
**SMS from friends**	7(7.4)	0
**Subscribed SMS service**	5(5.3)	0
**Family and friends**	12(12.8)	8(10.3)
**Health agencies**	19(20.2)	17(21.8)

* Information were obtained in late March/early April in 2013, 2–3 months after the first reported human H7N9 case. Multiple selection of information source was allowed.

SMS, short message service

The information sought by participants most was prevention of H7N9 (78.2%), transmission routes (70.5%), safe consumption of eggs/chicken (42.3%) and overall situation of the outbreak (32.0%).

### Outbreak information dissemination

The majority of participants thought H7N9 outbreak information was released in a transparent and timely manner. All participants in Beijing and Lanzhou and the majority of the Hangzhou participants commented that the outbreak information was released quickly and updated frequently. For example, one participant said, “Sometimes new patients were just found in the morning and the TV news reported it in the afternoon.” In addition, participants across groups praised H7N9 communication as transparent by comparing it with the communication response to SARS. “This time is much better than SARS, no information was hidden.”

Participants from the site with high prevalence of human infection with H7N9 virus had higher expectations of timely announcements of the emergence of the disease. Two participants from Hangzhou criticized the delay of the first announcement of the outbreak. One of them stated, “I do not think the government announced the disease in time. We first heard about the disease at the end of March, but the patients were hospitalized and even died almost one month earlier. It should have been announced earlier. This reminds me of SARS, when intentional underreporting was not uncommon. Who knows how many H7N9 patients have really been found …”

### Outbreak information needs

When asking what information released by health agencies was most helpful, almost all participants mentioned preventive methods. “The information telling us how to protect ourselves from getting infected is most helpful, such as washing hands more frequently, avoiding direct contact with birds and chickens, and things like that.” Some participants also valued the information about the evolving outbreak trends which helped them assess the disease severity. “I paid very close attention to the overall outbreak situation, it helped me to judge if the disease was spreading very quickly just like SARS did.” Some participants expressed their appreciation for information about H7N9 transmission routes. One said, “When I heard that H7N9 does not transmit from person to person, I felt so relieved.” Another explained, “When I heard that the majority of the patients had close contact with poultry, I just felt very relieved, I was sure I would not get the disease because I never directly touch chickens or ducks at all ...”

Some participants indicated their interest in knowing more about the clinical treatment status of the confirmed cases. “I want to know how many patients have died, how severe their symptoms are, and if there is effective medication that could cure this disease.”

### Perception of information from government

Participants perceived government agency information sources to be trustworthy. The most trusted information channels reported by the participants included China Central Television, major web portals, national and local newspapers, local TV channels, community information boards (which are typically located in the centre of residential communities and regularly updated by the community committee) and health education materials (posters and pamphlets) disseminated by health authorities. Participants had different comments on the credibility of social media; social media was more acceptable to young participants. Some participants recalled that much of the H7N9 information received had been via microblogs and SMS, and they described the information as “spurious and anecdotal.” Some young participants suggested the government should use more social media to release health messages. One participant said, “I want to recommend sending disease information more frequently via microblogging and WeChat. We young people almost always have the mobile phone in hand; it is very convenient for us [to get health messages].”

### Acceptability of the health recommendations

Most of the participants reported that all of the public health recommendations they received were clear and easy to follow. The concern for being infected by the H7N9 virus resulted in some behaviour changes, either by forming new behaviours or improving current behaviours. For example, one participant said, “All recommendations such as [open the windows] to air your room, wash hands, etc., are very easy to do. Actually I do these things almost every day, but since the start of this bird flu, I have been washing my hands more frequently and more carefully. I also remember to wash hands after touching raw eggs. I did not have this kind of habit before.”

However, one Hangzhou participant complained that the handwashing message was not practical, he said, “… to be honest, I cannot do that six-step-hand-washing process. I cannot memorize all those steps plus it wastes a lot of time. I do not think it will make a lot of difference from the way I wash my hands.”

### Factors affecting participants’ anxiety level

#### Disease severity

The severity and consequences of H7N9 infections were factors effecting people’s anxiety level. Some of the participants indicated that they were scared because the majority of the reported H7N9 cases were either in critical condition or deceased.

#### Distance from the outbreak sources

Although many Lanzhou participants reported that they never felt worried about the H7N9 outbreak since “It is very far [away from Lanzhou],” the majority of the participants in the other two cities felt worried during the first 2–3 weeks of the outbreak, but the anxiety eased afterwards. The rest of the participants from Beijing and Hangzhou reported that they “were not worried at all.”

#### Media coverage

The high intensity of media coverage about H7N9 in the early stage of the outbreak made the public vigilant and concerned about the situation. One participant said, “At the very beginning when I heard there were H7N9 patients, I did not think too much about it, but later when there were more and more media started reporting about this issue, I started worrying. It reminded me of SARS. It is scary if the situation cannot be controlled …”

However, when the media coverage decreased, people interpreted it as a sign of that the outbreak was contained. “I was worried at the beginning; however, recently I noticed that less media report this event, so I think this is not a big deal anymore. It must already be controlled.”

#### Transmission route of H7N9

The Chinese health authorities stated that there had been no proof of human-to-human transmission for H7N9, which successfully eased people’s anxiety. “Right now, I do not worry at all, because we cannot get this bird flu from people, I do not touch any live birds, chickens or ducks.”

#### Trust in government’s competence

The participants who “do not worry at all” indicated it was their trust in the government’s competency that made them worry-free: “We all know that China is more developed and stronger now. I am quite positive that our government absolutely has the capability to control this disease.”

### Recommendations for communication practice

One focus group in each city had full satisfaction with the H7N9 communication response by the Chinese government. The other groups suggested that public health recommendations be more specific and practical. As one participant stated, “We were just told to wear masks when going to crowded places but we do not know what kind of mask works for this disease. Do we need to wear N95 masks?”

One participant expressed his strong desire for more information about clinical treatment status. “There is very limited information about treatment. I want to know if there are any serious consequences for the survivors.”

Some participants complained that there was a lack of credible inquiry channels to seek help. One Hangzhou participant said, “My neighbour has some pigeons at his home, and they fly around. I wanted to know if these birds are dangerous, but I do not know where to go for this question.”

## Discussion

The goal of risk communication is to provide useful, relevant, accurate and needed information for a particular audience to make informed decisions about the risks they encounter. (*16*, *17*) Our results indicated that the majority of the participants felt their information needs were met concerning preventive measures, transmission routes and the evolving trends of the outbreak.

Trust is the cornerstone of effective risk communication. Being open, transparent and timely in communication will help to earn the trust of the audience. (*18*, *19*) Many participants commented that the H7N9 outbreak information was released and updated in a transparent and timely manner. However, people in epidemic areas might have much higher expectations for the timely release of information. The one-month time window between hospitalization and announcement of the first human H7N9 case made people question the timeliness of the information release and the openness of the government. More information about why the first announcement was not made earlier would have been helpful to avoid suspicion.

Normally when there is a health emergency, the information people need most is about preventive measures; (*20*) the same information need was reported by the participants. Similar to a previous study, (*21*) the participants had strong information needs about clinical treatment that was not sufficiently provided. Participants also requested more specific and practical public health recommendations. This is similar to Vaughan’s study that instructions for personal protective equipment usage should be clear and workable. (*3*) To dispel ambiguity, fill information gaps and increase compliance to public health recommendations, more efforts should be taken to collect public feedback on the recommendations to make them specific, feasible and clear.

In a 2014 study, (*22*) the majority of the participants stated that they believed government agencies had the capability to control the H7N9 outbreak and regarded official information sources as the most credible ones. While successful risk communication should motivate appropriate self-protective behaviour, (*3*) the general population’s acceptance of behavioural advice is strongly influenced by perceptions of integrity, credibility and competency of the authority. (*23*) The majority of the participants reported that they followed the public health recommendations to wash hands, open the windows more frequently, thoroughly cook food and avoid direct contact with poultry and wild birds. (*24*)

Our results suggest that the intensity of the media coverage is proportional to the public’s anxiety level about the reported health risk, similar to a study from the United States of America. (*25*) At the beginning of the outbreak, high mass media coverage about H7N9 successfully gained public attention. Mass media plays a large role in communicating health risks to the public, (*26*) and the participants in this study indicated that mass media were the major channels to receive H7N9 information. Mass media also could be used to disseminate public health recommendations to the population. Internet-based channels, including web portals and social media, were reported as preferences among the young population. Although the Chinese online community’s reaction to H7N9 was profound, (*27*) young participants still urged the government to communicate H7N9 information via social media. Health agencies may consider having an official presence on social media and using it routinely for health information delivery other than in emergencies so as to effectively reach the younger population.

Some participants reported they had difficulties in finding information to address their concerns or questions. A stronger two-way communication strategy should be applied. This helps to provide channels for the public to obtain specific information that they are concerned about and avoid misconceptions and rumours.

There were several limitations to our study. The results might not be representative as the participants were convenience sampled and were female dominant. The qualitative data analysis was done by only one researcher and later discussed with a team of researchers which might potentially limit the validity of the results. Although the moderator of the focus group is skillful and experienced, his governmental agency title might have caused some participants to hesitate in criticizing the government’s communication. Nevertheless, participants were open and felt comfortable enough to suggest how to improve the risk communication response.

## Conclusion

In conclusion, the majority of the focus group participants were satisfied with the Chinese health agency risk communication response to the 2013 H7N9 outbreak. They appreciated the transparent and timely information release and felt that their information needs had been met. Although some participants felt that the public health recommendations lacked feasibility and were not specific or clear enough, many participants reported behaviour change that conformed to public health recommendations. Social media should be more broadly used during public health emergencies to better reach the young population. Two-way inquiry channels, such as public health hotlines, should be more accessible to the public to help address questions, dispel rumours and clarify misunderstandings.
